# Hospitalization due to pneumonia in Australia, England, and Wales: An ecological cross-sectional study

**DOI:** 10.1097/MD.0000000000042163

**Published:** 2025-04-11

**Authors:** Mohammed Samannodi

**Affiliations:** a Department of Medicine, College of Medicine, Umm Al-Qura University, Makkah, Saudi Arabia.

**Keywords:** admission, Australia, England, hospitalization, pneumonia, Wales

## Abstract

Pneumonia and other lower respiratory tract diseases rank as the fourth leading cause of death worldwide. The objective of this study was to examine pneumonia hospitalization patterns, and trends in total pneumonia hospitalization stratified by age group, by type of hospitalization, and by age group in Australia, England, Wales. This study utilized 3 databases to obtain hospital admissions data: the National Hospital Morbidity Database for Australian hospital admissions data, the Hospital Episode Statistics database (HES) for England hospital admissions data, and the Patient Episode Database for Wales. Pneumonia hospitalization data were extracted utilizing J12 to J18 codes. From 2013 to 2020, there were 4,514,444 cases of pneumonia hospitalizations reported in Australia (646,515 [14.32%]), England (3,668,106 [81.25%]), and Wales (199,823 [4.43%]). The most common type of pneumonia hospitalization in Australia, England, and Wales was “pneumonia, organism unspecified,” accounting for 77.12%, 95.49%, and 95.75% of the total number of pneumonia hospitalizations in each country, respectively. The most common subtype of pneumonia hospitalization in Australia was “pneumonia, unspecified,” accounting for 72.98% of the total number of pneumonia hospitalizations in the country. The most common type of pneumonia hospitalization in England and Wales was “lobar pneumonia, unspecified,” accounting for 59.00% and 56.73% of the total number of pneumonia hospitalizations in each country, respectively. Most pneumonia hospitalizations in Australia, England, and Wales were non-same-day hospitalizations, accounting for 90.78%, 99.91%, and 99.95%, respectively. Pneumonia hospitalizations in Australia, England, and Wales were directly related to age. Males accounted for most pneumonia hospitalizations in Australia, England, and Wales. This study highlighted that hospitalization rate for pneumonia increased during the past decade in Australia, England, and Wales. The age and male gender were clearly contributing factors that affected pneumonia hospitalizations rate. Educational campaign aiming to increase public knowledge of pneumonia, its risk factors, and lifestyle modification should be prioritized to decrease pneumonia episodes.

## 1. Introduction

Pneumonia is classified by the World Health Organization (WHO) as an acute respiratory illness that impacts the oxygenation and lung parenchyma.^[1]^ Pneumonia and other lower respiratory tract diseases rank as the fourth leading cause of death worldwide.^[2]^ The incidence of pneumonia varies by demographics, health-care settings, and geographic region.^[3]^ Nonetheless, audits conducted by the British Thoracic Society indicated that the 30-day mortality rate has been declining since 2009 in the United Kingdom (UK).^[3]^

Furthermore, an increase in pneumonia-related hospitalizations have been observed in the Netherlands, Denmark, and the United States and more hospital admissions and bed days are caused by pneumonia than by any other respiratory illness in the UK.^[3–8]^ Besides, pneumonia causes 29,000 deaths annually in the UK, making it the third main cause of lung disease-related deaths, after lung cancer (the leading cause) and chronic obstructive pulmonary disease (COPD), which is the second leading cause.^[9]^ In addition, the UK has the 21st age-standardized pneumonia mortality rate out of 99 countries. Besides, pneumonia has a significant effect on the health-care systems of the UK and Europe since it is linked to high hospital admission and length of stay rates.^[9]^

There is an absence of comprehensive data regarding the epidemiology of pneumonia in Australia.^[10]^ Over 65,000 patients with this primary diagnosis visit Australian hospitals each year.^[11]^ However, there is a dearth of information regarding the etiology of pneumonia from an Australian perspective, as well as the number of individuals who present to hospitals with the illness but are not later admitted.^[11]^ Previous studies in Australia and the UK examined hospitalization profile due to respiratory diseases and demonstrated rising trends over the past 2 decades.^[12–14]^ There hasn’t been much advancement in the pathogenesis, epidemiology, or treatment of pneumonia.^[9]^ In fact, pneumonia ranked just 20 out of 25 infectious diseases in a study of UK funding for infectious disease research from 1997 to 2013, receiving little investment in relation to its disease burden.^[9]^ Therefore, the objective of this study was to examine pneumonia hospitalization patterns, and trends in total pneumonia hospitalization stratified by age group, by type of hospitalization, and by age group in Australia, England, Wales. Examining hospitalization profile due to pneumonia across these 3 countries provide insights for targeted health interventions and health-care policy developments across countries with different health-care system and policies.

## 2. Methods

### 2.1. Data sources

#### 2.1.1. Hospital admissions data

This study utilized 3 databases to obtain hospital admissions data: the National Hospital Morbidity Database (NHMD) for Australian hospital admissions data, the Hospital Episode Statistics database (HES) for England hospital admissions data, and the Patient Episode Database for Wales (PEDW). The NHMD, managed by the Australian Institute of Health and Welfare, contains patient episode-level records from private and public hospitals across Australia.^[15,16]^ HES and PEDW record all hospital admissions at all National Health Service hospitals. The quality of the 3 databases is constantly evaluated to ensure their accuracy and reliability.^[17–19]^ These databases were previously used to examine different health conditions across different age groups.^[20–23]^

#### 2.1.2. Population data

Population data were collected from 2 official sources: the Australian Bureau of Statistics for the national Australian population^[24,25]^ and the Office for National Statistics for the national England and Wales population.^[26]^ Mid-year population data were collected between 2013 and 2020.

### 2.2. Study population

This study included all publicly available pneumonia hospitalization data from the 3 databases for the period between 2013 and 2020.^[11,27,28]^ Pneumonia hospitalization data were extracted utilizing J12-J18 ICD-10 codes (“International Statistical Classification of Diseases and Related Health Problems 10th Revision”). The ICD codes used in this study were checked and verified by infectious diseases consultant.

### 2.3. Statistical analysis

The Statistical Package for Social Science Software (Chicago), version 29 was used to analyze the data for this study. Categorical variables were presented as frequencies and percentages. Hospitalisation rates were presented with 95% CIs. Pearson χ2 test of independence was used to examine the difference in admission rates between 2013 and 2020.

## 3. Results

### 3.1. Pneumonia hospitalization patterns

From 2013 to 2020, there were 4,514,444 cases of pneumonia hospitalizations reported in Australia (646,515 [14.32%]), England (3,668,106 [81.25%]), and Wales (199,823 [4.43%]). The most common type of pneumonia hospitalization in Australia, England, and Wales was “pneumonia, organism unspecified,” accounting for 77.12%, 95.49%, and 95.75% of the total number of pneumonia hospitalizations in each country, respectively. The most common subtype of pneumonia hospitalization in Australia was “pneumonia, unspecified,” accounting for 72.98% of the total number of pneumonia hospitalizations in the country. The most common type of pneumonia hospitalization in England and Wales was “lobar pneumonia, unspecified,” accounting for 59.00% and 56.73% of the total number of pneumonia hospitalizations in each country, respectively (Table 1).

**Table 1:** Pneumonia hospitalization and percentage from total admissions per ICD code.

ICD code	Description	Total number of pneumonia hospitalizations (% from total)		
		Australia	England	Wales
J12	Viral pneumonia, not elsewhere classified	65,263 (10.09)	27,690 (0.75)	995 (0.50)
J12.0	Adenoviral pneumonia	2689 (0.42)	1405 (0.04)	49 (0.02)
J12.1	Respiratory syncytial virus pneumonia	16,102 (2.49)	8347 (0.23)	235 (0.12)
J12.2	Parainfluenza virus pneumonia	4977 (0.77)	3459 (0.09)	160 (0.08)
J12.3	Human metapneumovirus pneumonia	9046 (1.40)	3765 (0.10)	178 (0.09)
J12.8	Other viral pneumonia	10,310 (1.59)	3680 (0.10)	95 (0.05)
J12.9	Viral pneumonia, unspecified	22,139 (3.42)	7034 (0.19)	278 (0.14)
J13	Pneumonia due to Streptococcus pneumoniae	16,036 (2.48)	48,217 (1.31)	1940 (0.97)
J14	Pneumonia due to Haemophilus influenzae	10,800 (1.67)	15,255 (0.42)	1253 (0.63)
J15	Bacterial pneumonia, not elsewhere classified	51,763 (8.01)	69,114 (1.88)	3997 (2.00)
J15.0	Pneumonia due to Klebsiella pneumoniae	1827 (0.28)	10,494 (0.29)	487 (0.24)
J15.1	Pneumonia due to Pseudomonas	8633 (1.34)	18,866 (0.51)	830 (0.42)
J15.2	Pneumonia due to Staphylococcus	4910 (0.76)	7753 (0.21)	652 (0.33)
J15.3	Pneumonia due to Streptococcus, group B	169 (0.03)	661 (0.02)	33 (0.02)
J15.4	Pneumonia due to other streptococci	1959 (0.30)	8557 (0.23)	536 (0.27)
J15.5	Pneumonia due to *Escherichia coli*	1009 (0.16)	4979 (0.14)	317 (0.16)
J15.6	Pneumonia due to other Gram-negative bacteria	1987 (0.31)	4102 (0.11)	271 (0.14)
J15.7	Pneumonia due to Mycoplasma pneumoniae	9676 (1.50)	3916 (0.11)	205 (0.10)
J15.8	Other bacterial pneumonia	2366 (0.37)	3047 (0.08)	253 (0.13)
J15.9	Bacterial pneumonia, unspecified	19,227 (2.97)	6739 (0.18)	413 (0.21)
J16	Pneumonia due to other infectious organisms, not elsewhere classified	1658 (0.26)	1045 (0.03)	78 (0.04)
J16.0	Chlamydial pneumonia	808 (0.12)	102 (0.00)	5 (0.00)
J16.8	Pneumonia due to other specified infectious organisms	850 (0.13)	943 (0.03)	73 (0.04)
J17	Pneumonia in diseases classified elsewhere	2408 (0.37)	3986 (0.11)	228 (0.11)
J17.0	Pneumonia in bacterial diseases classified elsewhere	408 (0.06)	512 (0.01)	55 (0.03)
J17.1	Pneumonia in viral diseases classified elsewhere	98 (0.02)	282 (0.01)	5 (0.00)
J17.2	Pneumonia in mycoses	798 (0.12)	2086 (0.06)	117 (0.06)
J17.3	Pneumonia in parasitic diseases	1005 (0.16)	1060 (0.03)	51 (0.03)
J17.8	Pneumonia in other diseases classified elsewhere	99 (0.02)	46 (0.00)	0 (0.00)
J18	Pneumonia, organism unspecified	498,587 (77.12)	3,502,799 (95.49)	191,332 (95.75)
J18.0	Bronchopneumonia, unspecified	16,618 (2.57)	116,405 (3.17)	7679 (3.84)
J18.1	Lobar pneumonia, unspecified	4471 (0.69)	2,164,345 (59.00)	113,367 (56.73)
J18.2	Hypostatic pneumonia, unspecified	349 (0.05)	1269 (0.03)	72 (0.04)
J18.8	Other pneumonia, organism unspecified	5294 (0.82)	2710 (0.07)	107 (0.05)
J18.9	Pneumonia, unspecified	471,855 (72.98)	1,218,070 (33.21)	70,107 (35.08)

ICD = International Statistical Classification of Diseases.

### 3.2. Trends in total pneumonia hospitalization

**Figure 1.**
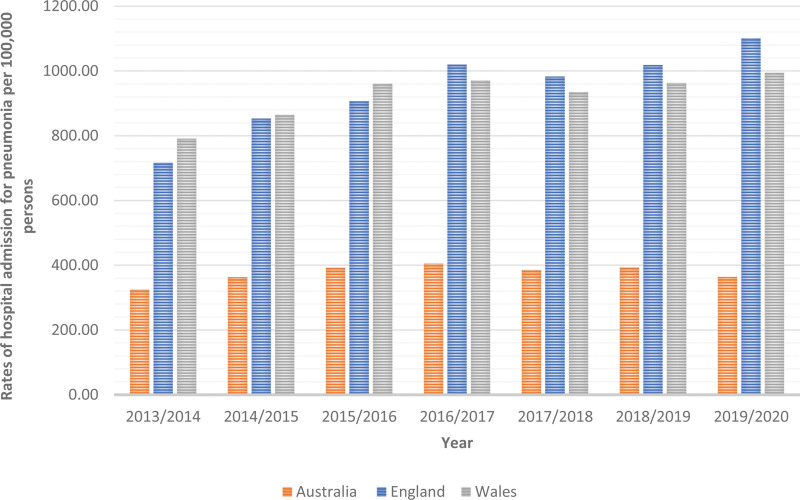
Total pneumonia hospitalization rates in Australia, England, and Wales from 2013 to 2020.

During the study period, the total number of pneumonia hospitalizations in the 3 countries increased by 52.03%, from 489,855 in 2013 to 744,722 in 2020; raised by 22.43% in Australia (from 76,206 to 93,299), by 59.39% in England (from 389,333 to 620,541), and by 27.00% in Wales (from 24,316 to 30,882). Still, the total pneumonia hospitalization rates in Australia increased by 12.06% from 324.62 (95% CI 322.32–326.92) per 100,000 persons in 2013 to 363.75 (95% CI 361.42–366.08) per 100,000 persons in 2020, (trend test, *P* < .001). The total pneumonia hospitalization rates in England increased by 53.85% from 716.08 (95% CI 713.84–718.32) per 100,000 persons in 2013 to 1101.70 (95% CI 1098.97–1104.42) in 2020, (trend test, *P* < .001). The total pneumonia hospitalization rates in Wales increased by 25.75% from 791.08 (95% CI 781.17–800.98) per 100,000 persons in 2013 to 994.76 (95% CI 983.72–1005.79) in 2020 (trend test, *P* < .001) (Fig. 1).

### 3.3. Trends in total pneumonia hospitalization stratified by hospitalization type

**Figure 2.**
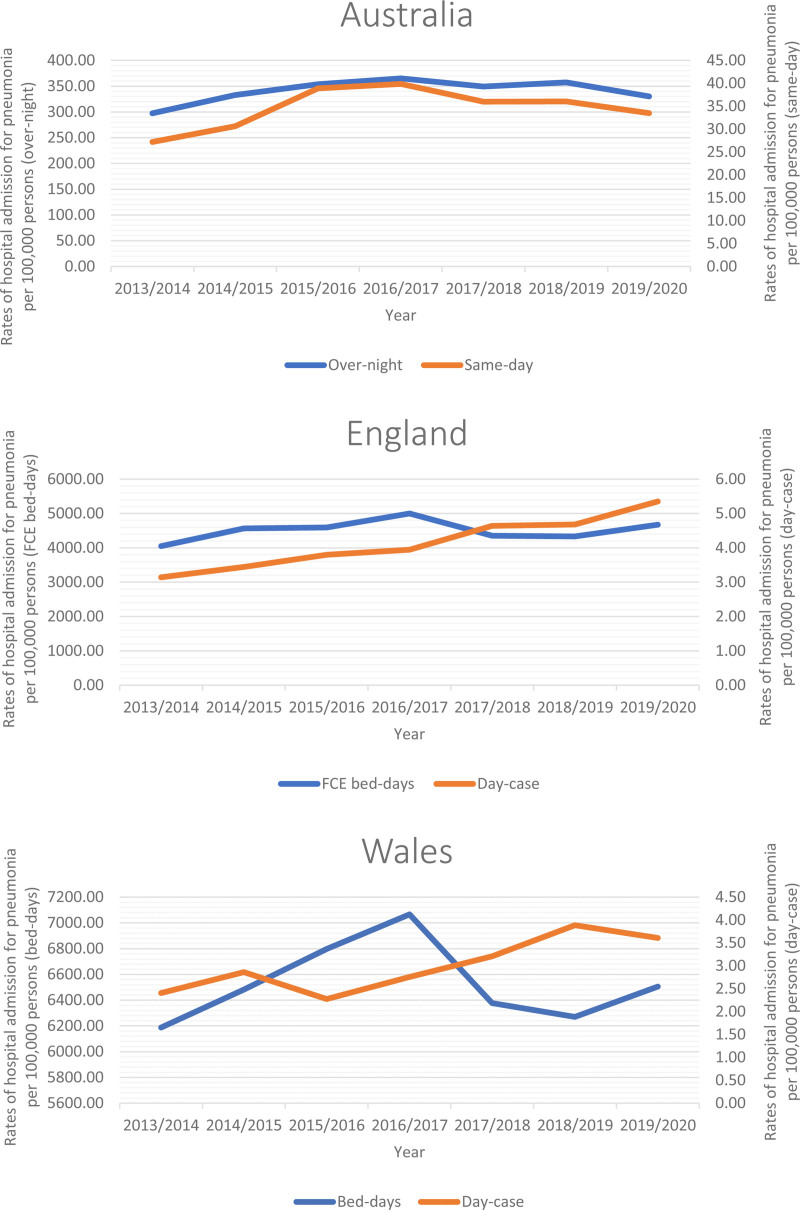
Total pneumonia hospitalization rates in Australia, England, and Wales from 2013 to 2020 stratified by hospitalization type.

During the study period, most pneumonia hospitalizations in Australia, England, and Wales were non-same-day hospitalizations, accounting for 90.78%, 99.91%, and 99.95%, respectively. The rates of same-day pneumonia hospitalization increased by 23.18%, 70.28%, and 49.85% in Australia, England, and Wales, respectively. On the other hand, the rates of non-same-day pneumonia hospitalization increased by 11.04%, 15.35%, and 5.14% in Australia, England, and Wales, respectively (Table 2 and Fig. 2).

**Table 2:** Percentage change in the total pneumonia hospitalization rates from 2013 to 2020 in Australia, England, and Wales stratified by hospitalization type.

Hospitalization type	Country	Hospitalization rates in 2013 per 100,000 persons (95% CI)	Hospitalization rates in 2020 per 100,000 persons (95% CI)	% change from 2013 to 2020
Same d	Australia	27.20 (26.53–27.87)	33.50 (32.79–34.21)	23.18
	England	3.14 (2.99–3.29)	5.35 (5.16–5.54)	70.28
	Wales	2.41 (1.86–2.96)	3.61 (2.94–4.28)	49.85
Non-same d	Australia	297.42 (295.22–299.62)	330.25 (328.03–332.47)	11.04
	England	4048.83 (4043.59–4054.07)	4670.16 (4664.65–4675.67)	15.35
	Wales	6187.64 (6160.71–6214.58)	6505.46 (6478.03–6532.90)	5.14

### 3.4. Trends in total pneumonia hospitalization stratified by age group

**Figure 3.**
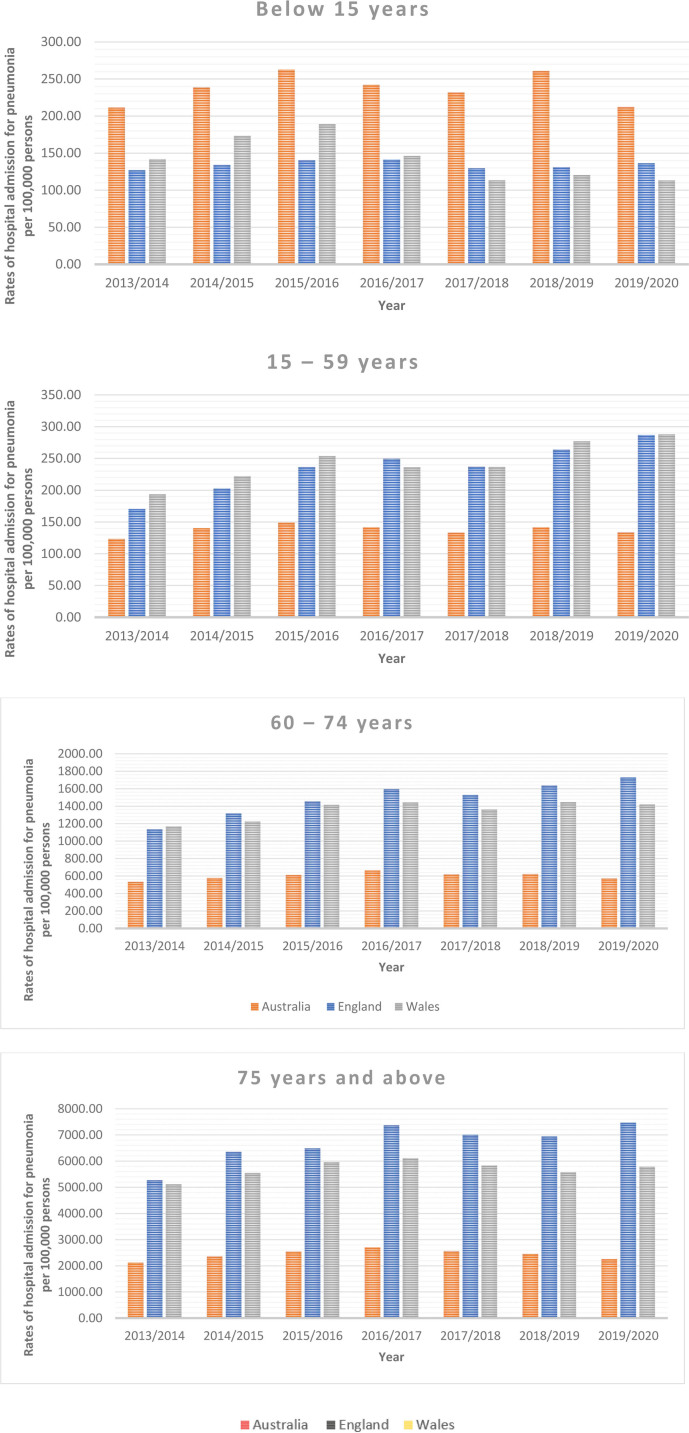
Total pneumonia hospitalization rates in Australia, England, and Wales from 2013 to 2020 stratified by age group.

From 2013 to 2020, pneumonia hospitalizations in Australia, England, and Wales were directly related to age. The pneumonia hospitalization rates increased among all age groups in Australia, England, and Wales, except for the age group below 15 years in Wales, where the rate decreased (Table 3 and Fig. 3).

**Table 3:** Percentage change in the total pneumonia hospitalization rates from 2013–2020 in Australia, England, and Wales stratified by age group.

Age group	Country	Hospitalization rates in 2013 per 100,000 persons (95% CI)	Hospitalization rates in 2020 per 100,000 persons (95% CI)	% change from 2013–2020
Below 15 yr	Australia	211.57 (207.29–215.84)	212.37 (208.23–216.50)	0.38
	England	126.92 (124.68–129.17)	136.31 (134.01–138.61)	7.40
	Wales	141.86 (131.60–152.12)	113.25 (104.08–122.42)	−20.17
15 to 59 yr	Australia	123.47 (121.65–125.29)	133.80 (131.97–135.63)	8.37
	England	170.43 (169.01–171.85)	287.04 (285.22–288.87)	68.42
	Wales	193.50 (187.00–200.00)	287.84 (279.87–295.82)	48.76
60 to 74 yr	Australia	533.23 (525.25–541.22)	574.35 (566.76–581.93)	7.71
	England	1135.36 (1128.05–1142.68)	1728.20 (1719.55–1736.85)	52.22
	Wales	1169.54 (1140.43–1198.65)	1419.20 (1388.04–1450.35)	21.35
75 yr and above	Australia	2124.80 (2101.80–2147.80)	2253.07 (2231.48–2274.67)	6.04
	England	5268.87 (5247.88–5289.85)	7468.76 (7445.23–7492.29)	41.75
	Wales	5113.15 (5030.80–5195.51)	5783.44 (5700.14–5866.75)	13.11

### 3.5. Trends in total pneumonia hospitalization stratified by gender

**Figure 4.**
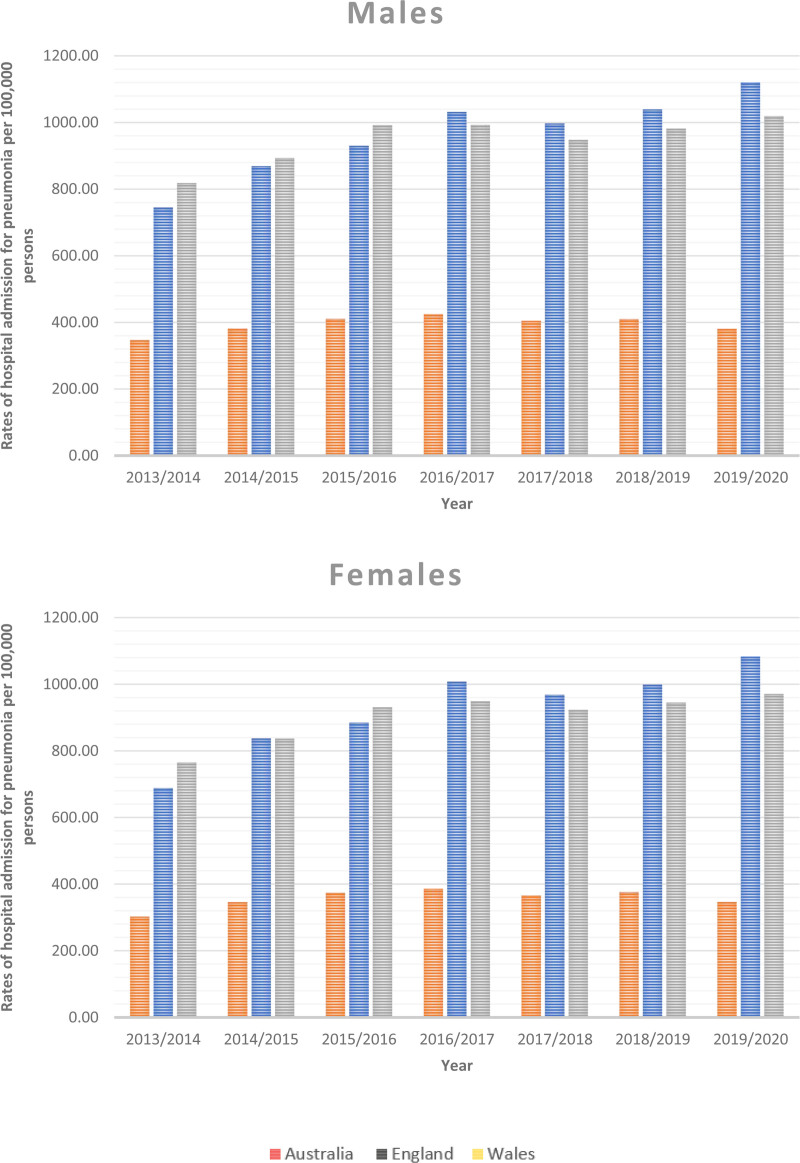
Total pneumonia hospitalization rates in Australia, England, and Wales from 2013 to 2020 stratified by gender.

Over the study period, males accounted for most pneumonia hospitalizations in Australia, England, and Wales. The pneumonia hospitalization rates among males increased by 9.86%, 50.41%, and 24.62% in Australia, England, and Wales, respectively. Similarly, the pneumonia hospitalization rates among females increased by 14.56%, 57.39%, and 26.90% in Australia, England, and Wales, respectively (Table 4 and Fig. 4).

**Table 4:** Percentage change in the total pneumonia hospitalization rates from 2013 to 2020 in Australia, England, and Wales stratified by gender.

Gender	Country	Hospitalization rates in 2013 per 100,000 persons (95% CI)	Hospitalization rates in 2020 per 100,000 persons (95% CI)	% change from 2013 to 2020
Males	Australia	346.77 (343.40–350.15)	380.95 (377.57–384.34)	9.86
	England	744.88 (741.62–748.14)	1120.38 (1116.45–1124.30)	50.41
	Wales	817.77 (803.40–832.14)	1019.11 (1003.15–1035.07)	24.62
Females	Australia	302.72 (299.59–305.86)	346.79 (343.59–350.00)	14.56
	England	688.15 (685.07–691.23)	1083.10 (1079.31–1086.88)	57.39
	Wales	765.32 (751.66–778.98)	971.22 (955.94–986.50)	26.90

### 3.6. Trends in pneumonia hospitalization

The highest change in hospitalization rate in Australia was observed for bacterial pneumonia, not elsewhere classified related hospitalization. However, in England and Wales the highest change in hospitalization rate in was observed for viral pneumonia-related hospitalization. For further details on the change in pneumonia hospitalization rates from 2013 to 2020 in Australia, England, and Wales, refer to Table 5.

**Table 5:** Percentage change in pneumonia hospitalization rates from 2013 to 2020 in Australia, England, and Wales per ICD code.

ICD code	Australia			England			Wales		
	Hospitalization rates in 2013 per 100,000 persons (95% CI)	Hospitalization rates in 2020 per 100,000 persons (95% CI)	% change from 2013 to 2020	Hospitalization rates in 2013 per 100,000 persons (95% CI)	Hospitalization rates in 2020 per 100,000 persons (95% CI)	% change from 2013 to 2020	Hospitalization rates in 2013 per 100,000 persons (95% CI)	Hospitalization rates in 2020 per 100,000 persons (95% CI)	% change from 2013 to 2020
J12	21.58 (20.99–22.17)	46.72 (45.88–47.56)	116.49%	3.12 (2.98–3.27)	14.73 (14.42–15.05)	371.50%	2.08 (1.57–2.59)	9.34 (8.27–10.42)	348.64
J12.0	0.86 (0.75–0.98)	1.32 (1.18–1.46)	52.84%	0.18 (0.14–0.22)	0.65 (0.58–0.72)	261.49%	0.03 (−0.03 to 0.10)	0.90 (0.57–1.24)	2672.32
J12.1	5.29 (5.00–5.59)	10.39 (10.00–10.79)	96.31%	0.49 (0.43–0.55)	5.97 (5.77–6.17)	1116.18%	0.49 (0.24–0.73)	2.83 (2.24–3.43)	480.87
J12.2	1.32 (1.17–1.46)	4.51 (4.25–4.77)	242.70%	0.45 (0.39–0.50)	1.55 (1.45–1.65)	246.39%	0.23 (0.06–0.40)	1.39 (0.97–1.80)	508.21
J12.3	2.17 (1.98–2.36)	6.61 (6.30–6.93)	204.37%	0.42 (0.36–0.47)	1.81 (1.70–1.92)	334.16%	0.16 (0.02–0.31)	1.64 (1.19–2.09)	909.91
J12.8	2.61 (2.40–2.81)	10.23 (9.84–10.62)	292.27%	0.35 (0.30–0.40)	2.10 (1.98–2.22)	503.17%	0.20 (0.04–0.35)	1.22 (0.83–1.61)	527.07
J12.9	9.32 (8.93–9.72)	13.65 (13.20–14.11)	46.42%	1.24 (1.15–1.34)	2.65 (2.52–2.79)	113.65%	0.98 (0.63–1.33)	1.35 (0.94–1.76)	38.62
J13	7.98 (7.62–8.34)	9.74 (9.36–10.12)	22.07%	9.81 (9.55–10.07)	14.65 (14.33–14.96)	49.28%	6.96 (6.03–7.89)	9.24 (8.18–10.31)	32.79
J14	4.96 (4.67–5.24)	5.10 (4.82–5.38)	2.85%	3.44 (3.28–3.60)	3.85 (3.69–4.02)	12.07%	5.30 (4.49–6.12)	4.61 (3.85–5.36)	−13.14
J15	17.99 (17.45–18.54)	47.71 (46.86–48.55)	165.13%	15.14 (14.81–15.46)	19.20 (18.83–19.56)	26.81%	22.42 (20.74–24.09)	15.59 (14.20–16.98)	−30.45
J15.0	1.09 (0.96–1.23)	0.99 (0.86–1.11)	−9.90%	2.42 (2.29–2.55)	2.91 (2.77–3.05)	20.16%	3.03 (2.41–3.64)	2.93 (2.33–3.53)	−3.12
J15.1	4.51 (4.24–4.78)	4.69 (4.42–4.95)	3.88%	4.47 (4.29–4.65)	5.25 (5.06–5.44)	17.45%	4.55 (3.80–5.31)	2.74 (2.16–3.32)	−39.89
J15.2	2.90 (2.68–3.11)	2.28 (2.10–2.47)	−21.26%	1.99 (1.87–2.10)	1.81 (1.70–1.92)	−8.92%	4.20 (3.47–4.92)	2.16 (1.64–2.67)	−48.58
J15.3	0.08 (0.04–0.11)	0.09 (0.05–0.12)	11.86%	0.14 (0.11–0.17)	0.15 (0.11–0.18)	6.96%	0.07 (−0.03 to 0.16)	0.00 (0.00–0.00)	−100.00
J15.4	1.07 (0.94–1.20)	1.07 (0.94–1.19)	−0.09%	1.78 (1.67–1.89)	2.28 (2.15–2.40)	28.04%	2.54 (1.97–3.10)	2.61 (2.04–3.18)	2.82
J15.5	0.59 (0.49–0.69)	0.51 (0.42–0.60)	−13.12%	1.11 (1.02–1.19)	1.24 (1.15–1.33)	12.43%	2.18 (1.66–2.70)	0.68 (0.39–0.97)	−68.97
J15.6	0.83 (0.71–0.94)	1.26 (1.12–1.40)	52.39%	0.72 (0.65–0.79)	1.23 (1.14–1.32)	71.52%	0.78 (0.47–1.09)	1.19 (0.81–1.58)	52.64
J15.7	3.34 (3.11–3.57)	6.31 (6.00–6.62)	89.01%	0.60 (0.54–0.67)	1.60 (1.50–1.70)	165.97%	0.68 (0.39–0.98)	1.84 (1.36–2.31)	168.74
J15.8	1.08 (0.95–1.22)	1.31 (1.17–1.45)	21.43%	0.80 (0.72–0.87)	0.58 (0.52–0.65)	−26.83%	1.53 (1.09–1.97)	0.74 (0.44–1.04)	−51.55
J15.9	2.51 (2.31–2.71)	29.20 (28.54–29.86)	1063.89%	1.12 (1.03–1.21)	2.15 (2.03–2.27)	91.32%	2.86 (2.26–3.46)	0.71 (0.41–1.00)	−75.25
J16	0.93 (0.81–1.06)	0.00 (0.00–0.00)	−100.00%	0.22 (0.18–0.25)	0.42 (0.36–0.47)	93.06%	0.33 (0.12–0.53)	0.48 (0.24–0.73)	48.52
J16.0	0.45 (0.37–0.54)	0.00 (0.00–0.00)	−100.00%	0.03 (0.02–0.05)	0.03 (0.02–0.05)	7.88%	0.00 (0.00–0.00)	0.10 (−0.01 to 0.21)	-
J16.8	0.48 (0.39–0.57)	0.00 (0.00–0.00)	−100.00%	0.18 (0.15–0.22)	0.38 (0.33–0.43)	107.54%	0.33 (0.12–0.53)	0.39 (0.17–0.61)	18.81
J17	0.98 (0.85–1.10)	1.98 (1.80–2.15)	102.64%	1.01 (0.93–1.10)	0.94 (0.86–1.02)	−6.81%	1.27 (0.87–1.67)	1.06 (0.70–1.43)	−16.22
J17.0	0.35 (0.27–0.42)	0.12 (0.08–0.17)	−63.84%	0.31 (0.27–0.36)	0.01 (0.00–0.02)	−95.46%	0.55 (0.29–0.82)	0.00 (0.00–0.00)	−100.00
J17.1	0.00 (0.00–0.00)	0.09 (0.05–0.13)	-	0.09 (0.06–0.11)	0.05 (0.03–0.07)	−41.68%	0.03 (−0.03 to 0.10)	0.00 (0.00–0.00)	−100.00
J17.2	0.23 (0.16–0.29)	1.70 (1.54–1.86)	651.20%	0.36 (0.31–0.41)	0.50 (0.44–0.56)	36.50%	0.52 (0.27–0.78)	0.55 (0.29–0.81)	5.20
J17.3	0.40 (0.32–0.49)	0.00 (0.00–0.00)	−100.00%	0.24 (0.20–0.28)	0.36 (0.31–0.41)	49.58%	0.16 (0.02–0.31)	0.52 (0.26–0.77)	216.84
J17.8	0.00 (0.00–0.00)	0.07 (0.03–0.10)	-	0.01 (0.00–0.01)	0.02 (0.01–0.03)	253.94%	0.00 (0.00–0.00)	0.00 (0.00–0.00)	-
J18	270.20 (268.10–272.30)	252.51 (250.57–254.45)	−6.55%	683.34 (681.15–685.53)	1047.91 (1045.25–1050.57)	53.35%	752.72 (743.06–762.38)	954.43 (943.61–965.24)	26.80
J18.0	11.77 (11.33–12.21)	6.56 (6.24–6.87)	−44.30%	31.87 (31.40–32.35)	26.32 (25.90–26.75)	−17.41%	42.75 (40.44–45.06)	25.83 (24.05–27.62)	−39.57
J18.1	2.70 (2.49–2.91)	1.67 (1.51–1.83)	−37.97%	422.49 (420.77–424.22)	647.09 (644.99–649.18)	53.16%	469.55 (461.91–477.19)	549.98 (541.75–558.21)	17.13
J18.2	0.29 (0.22–0.36)	0.11 (0.07–0.15)	−60.97%	0.37 (0.32–0.42)	0.30 (0.26–0.35)	−18.76%	0.39 (0.17–0.61)	0.58 (0.31–0.85)	48.52
J18.8	3.27 (3.04–3.50)	2.50 (2.31–2.69)	−23.51%	0.72 (0.65–0.79)	0.67 (0.61–0.74)	−6.43%	0.33 (0.12–0.53)	0.55 (0.29–0.81)	68.32
J18.9	252.17 (250.14–254.20)	241.67 (239.77–243.57)	−4.17%	227.88 (226.61–229.15)	373.52 (371.93–375.12)	63.91%	239.70 (234.24–245.17)	377.49 (370.66–384.31)	57.48

ICD = International Statistical Classification of Diseases.

## 4. Discussion

This study aimed to examine pneumonia hospitalization patterns in Australia, England, Wales during the past 2 decades. In this study, the most common type of pneumonia hospitalization in Australia, England, and Wales was “pneumonia, organism unspecified,” accounting for 77.12%, 95.49%, and 95.75% of the total number of pneumonia hospitalizations in each country, respectively. Besides, the most common subtype of pneumonia hospitalization in Australia was “pneumonia, unspecified,” accounting for 72.98% of the total number of pneumonia hospitalizations in the country. The most common type of pneumonia hospitalization in England and Wales was “lobar pneumonia, unspecified,” accounting for 59.00% and 56.73%. In a large study conducted in England using the Hospital Episode Statistics databased on all pneumonia episodes that reported between April 1997 and March 2005, around 42% of pneumonia hospital admissions was related to nonspecific pneumonia, influenza for 10%, H. influenzae for 9%, Bordetella pertussis for 7%, and RSV for 5%.^[29]^ On the other hands, all-cause pneumonia accounted for 791,812 admissions in Australian hospitals between July 1998 and June 2011. Pneumococcal and lobar pneumonia episodes made up 4.3% of the total number of admissions, while other specific causal organism codes accounted for the remaining 10.0%.^[30]^ A basic issue with pneumonia is the inadequate classification of related but heterogeneous diseases and clinical phenotypes due to the lack of generally recognized, widely used, and acceptable definition or definitions. The widespread incapacity to identify the infectious organism or organisms causing lung infections, which calls for empirical antibiotic therapy, is the clearest indication of the severity of this issue.^[31,32]^

In this study, the total pneumonia hospitalization rates in Australia increased by 12.06% from 324.62 (95% CI 322.32–326.92) per 100,000 persons in 2013 to 363.75 (95% CI 361.42–366.08) per 100,000 persons in 2020, (trend test, *P* < .001). The total pneumonia hospitalization rates in England increased by 53.85% from 716.08 (95% CI 713.84–718.32) per 100,000 pers persons in 2013 to 1101.70 (95% CI 1098.97–1104.42) in 2020, (trend test, *P* < .001). The total pneumonia hospitalization rates in Wales increased by 25.75% from 791.08 (95% CI 781.17–800.98) per 100,000 persons in 2013 to 994.76 (95% CI 983.72–1005.79) in 2020 (trend test, *P* < .001). Early research in the US and Europe found that hospitalizations for pneumonia among adults were on the rise over time with the aging of the population and the rising incidence of underlying diseases being 2 potential risk factors.^[4–6]^ Comorbidities, excessive alcohol consumption, and smoking are other risk factors that increase the possibility of pneumonia hospitalization.^[33]^ The all-cause pneumonia hospitalization rate per 1000 person-years among individuals aged 41 to 64, 65 to 79, and ≥ 80 years rose from 2.92, 14.06, and 31.71 in 1997 to 5.27, 21.32, and 60.38 in 2011, according to a study based on the entire Danish population.^[34]^ The percentage of hospitalized patients with pneumonia who also had underlying illnesses rose from 42.9% to 46.2% according to a previous US study that used data from the National Hospital Discharge Survey. Moreover, the rate of hospitalization for pneumonia among persons aged 65 and older increased by 20% between 1988 and 2002.^[4]^

Hospitalization, morbidity, and mortality rates can be reduced by identifying risk factors and putting policies in place to limit exposure to modifiable circumstances.^[35–40]^ Age,^[35,36]^ COPD,^[35]^ asthma, diabetes mellitus, congestive heart failure, smoking, malnutrition, and aspiration are risk factors linked to CAP.^[35,36,38,40]^ Additional research indicates that certain types of medication may raise the risk of CAP. According to a nested case-control study, older individuals who take atypical antipsychotic medications had a 2.1-fold increased risk of pneumonia.^[39]^ Proton pump inhibitors dramatically raise the incidence of hospital-acquired pneumonia by 30% and CAP by 50% to 89%.^[37]^ A higher incidence of pneumonia hospitalization was linked to advanced age, male gender, and prior underlying illnesses.^[41]^

In this study, most pneumonia hospitalizations in Australia, England, and Wales were non-same-day hospitalizations, accounting for 90.78%, 99.91%, and 99.95%, respectively. The rates of same-day pneumonia hospitalization increased by 23.18%, 70.28%, and 49.85% in Australia, England, and Wales, respectively. On the other hand, the rates of non-same-day pneumonia hospitalization increased by 11.04%, 15.35%, and 5.14% in Australia, England, and Wales, respectively. There are multiple factors influencing length of stay in pneumonia patients such as health-care system and hospital management, clinical practice such as (the abilities, expertise, and proficiency of doctors using sound, established clinical recommendations, Early transition from intravenous to oral antibiotic treatment, withdrawal of oxygen when necessary, physicians’ opinions regarding the efficacy of medical interventions), and patient characteristics.^[42]^ Furthermore, a large study included 400,000 adult patients done in (January 1998 to March 1999) in a public acute-care teaching hospital in Valencia (Spain) found that 29% of patients had possibly unnecessary hospitalization due to the “weekend effect” and/or delays in the availability of laboratory test results or chest radiography days.^[43]^ In addition, multiple clinical factors may affect duration of hospitalization like hypoxemia, anemia, neoplastic illness, and complications within 72 hours of admission.^[43]^ A common requirement for hospital admission is hypoxemia, which supports the admission patient to intensive care.^[44]^ It is crucial to note that hospitals with the shortest length of stay did not have higher readmission rates or post-discharge mortality rates among CAP patients.^[45,46]^ These statistics may serve as a helpful standard for doctors and hospitals to lower the length of stay for CAP patients without posing any new risk on the patients.

In this study, males accounted for most pneumonia hospitalizations in Australia, England, and Wales. The pneumonia hospitalization rates among males increased by 9.86%, 50.41%, and 24.62% in Australia, England, and Wales, respectively. Similarly, the pneumonia hospitalization rates among females increased by 14.56%, 57.39%, and 26.90% in Australia, England, and Wales, respectively. This result is consistent with large cohort study conducted in USA during 2006 through 2010, where males exhibited higher rates of at-risk behaviors and comorbidities,^[47]^ which probably contributes significantly to their increased prevalence of pneumonia. Overall, the age of the patients and the study locations may have had an impact on the sex differences observed in these cohorts in addition to other illnesses. It should come as no surprise that delayed hospital presentation for other acute medical conditions has been documented in females,^[48]^ and this may also be a factor in the lower incidence of CAP seen in females, who tend to seek hospital assistance only when clinical conditions worsen (rather than mild cases), which are likely to resolve on their own.^[48]^ Smoking and male gender have previously been shown to be risk factors for pneumonia due to Legionella pneumophila.^[49]^ Immune responses to particular pathogens and sex-specific immune responses interact during the course of an illness an obvious illustration of this link between the incidence and prognosis of influenza virus A infections by sex.^[50]^ The WHO released a paper in 2010 outlining the evidence that gender and sex should be taken into account when assessing influenza virus infection exposure and outcome.^[51]^

The highest change in hospitalization rate in Australia was observed for bacterial pneumonia, not elsewhere classified related hospitalization. However, in England and Wales the highest change in hospitalization rate in was observed for viral pneumonia-related hospitalization Streptococcus pneumoniae, Hemophilus influenzae (including H. influenzae type b-Hib), Staphylococcus aureus, and other bacteria are the culprits behind pneumonia. Among them include Mycobacterium tuberculosis, Chlamydia, and Mycoplasma.^[52]^ Moreover, influenza continues to be the clinically most important viral cause of adult CAP among viral diseases; other prevalent viral pathogens include Adenovirus, RSV, and parainfluenza viruses (PIV). Human metapneumovirus (HMPV), coronaviruses, and rhinoviruses are among the other viruses found in CAP patients.^[53]^

It worth mentioning that the COVID-19 pandemic control measures have had an impact on influenza and other respiratory virus transmission. Public health messaging, physical and social separation, national lockdowns, hand cleanliness, facial coverings, and travel limitations were some of these efforts. Some influenza indicators are also directly and indirectly impacted by changes in health-care-seeking behaviors.^[54]^ When interpreting the influenza surveillance indicators presented in this report, these factors remain crucial, particularly when comparing with previous seasons. Even that vaccination is still the most well-known and researched method of preventing pneumonia,^[55]^ it is crucial to understand the modifiable risk factors that contribute to the development of the illness. Malnutrition, which is a significant risk factor for pneumonia.^[40]^ Even in older people, the risk of pneumonia may be reduced by reducing lifestyle-related risk factors like smoking and alcohol use.^[52]^ Pneumonia risk can also be decreased by closely monitoring side effects and carefully reviewing prescription drugs.^[37–40]^ Furthermore, since pathogens were not found in most patients, this likely reflects the relative insensitivity of diagnostic techniques. It is necessary to continue developing new quick diagnostic technologies that can reliably identify and differentiate between possible pneumonia pathogens.^[56]^

This study has limitations. The study time frame was restricted to examine admissions until 2020. This was due to the fact that COVID-19 pandemic affected the normal pattern of admissions due to different health conditions. This study utilized publicly available data on the population level rather than on the individual level of the patients. This restricted the ability to examine the impact of confounding factors on the estimated hospitalization rates. Overestimation is a common limitation for this type of study as admission rates could include readmission and multiple admissions of the same patient. Therefore, the study findings should be interpreted carefully.

## 5. Conclusion

This study highlighted that hospitalization rate for pneumonia increased during the past decade in Australia, England, and Wales. The most common type of pneumonia hospitalization was “pneumonia, organism unspecified.” Most pneumonia hospitalizations were non-same-day hospitalizations. The age and male gender were clearly contributing factors that affected pneumonia hospitalizations rate. Future studies should identify modifiable risk factors among these population specifically and work on decreasing their impact. Educational campaign aiming to increase public knowledge of pneumonia, its risk factors, and lifestyle modification should be prioritized to decrease pneumonia episodes.

## Author contributions

**Conceptualization:** Mohammed Samannodi.

**Data curation:** Mohammed Samannodi.

**Formal analysis:** Mohammed Samannodi.

**Funding acquisition:** Mohammed Samannodi.

**Investigation:** Mohammed Samannodi.

**Methodology:** Mohammed Samannodi.

**Project administration:** Mohammed Samannodi.

**Resources:** Mohammed Samannodi.

**Software:** Mohammed Samannodi.

**Supervision:** Mohammed Samannodi.

**Validation:** Mohammed Samannodi.

**Visualization:** Mohammed Samannodi.

**Writing – original draft:** Mohammed Samannodi.

**Writing – review & editing:** Mohammed Samannodi.
